# Pharmacokinetics of a novel transdermal rivastigmine patch for the treatment of Alzheimer’s disease: a review

**DOI:** 10.1111/j.1742-1241.2009.02052.x

**Published:** 2009-05

**Authors:** A Kurz, M Farlow, G Lefèvre

**Affiliations:** 1 Department of Psychiatry and Psychotherapy, Technische Universität MünchenMunich, Germany; 2 Department of Neurology, Indiana University School of MedicineIndianapolis, IN, USA; 3 Novartis, Translational SciencesBasel, Switzerland

## Abstract

**Background:**

Cholinesterase inhibitors have all been available in oral formulations, but a rivastigmine transdermal patch has now been developed and is approved in many countries worldwide for the treatment of mild-to-moderate Alzheimer’s disease (AD) (including the USA, Latin America, Europe and Asia).

**Objectives:**

To review the available pharmacokinetic data that supported the rationale behind the development of the rivastigmine transdermal patch and its clinical effects in dementia therapy. This article will also discuss how the patch may alter the treatment paradigm for patients with AD.

**Results:**

The 9.5 mg/24 h rivastigmine patch was shown to provide comparable exposure to the highest recommended doses of capsules (12 mg/day) with significantly lower maximum plasma concentration (*C*_max_ 8.7 vs. 21.6 ng/ml) and slower absorption rate (*t*_max_ 8.1 vs. 1.4 h). In a clinical trial of 1195 AD patients, this translated into similar efficacy with three times fewer reports of nausea and vomiting (7.2% vs. 23.1%, and 6.2% vs. 17.0% respectively). Consequently, more patients in the 9.5 mg/24 h patch group achieved their target therapeutic dose at the end of the study, compared with those in the 12 mg/day capsule group (95.9% vs. 64.4%).

**Conclusion:**

The rivastigmine patch provides continuous drug delivery over 24 h and similar efficacy to the highest recommended dose of oral rivastigmine with improved tolerability. This may allow patients to achieve optimal therapeutic doses and to benefit from a longer duration of treatment.

**Review Criteria**

The information considered in this review was gathered from key publications comparing efficacy, pharmacokinetic and pharmacodynamic data for the rivastigmine transdermal patch and oral rivastigmine formulations. Clinical data were taken from the pivotal phase-III IDEAL trial. These data were analysed to review the potential benefits of patch therapy vs. oral therapy, and how this may alter the management of dementia patients in clinical practice.

**Message for the Clinic**

The rivastigmine transdermal patch provides smooth, continuous delivery of the drug over 24 h. This translates into similar efficacy to the highest recommended dose of oral rivastigmine with an improved tolerability profile. Fewer side effects would allow patients to achieve optimal therapeutic doses.

## Introduction

Cholinesterase inhibitors are widely used in the symptomatic treatment of Alzheimer’s disease (AD) in clinical practice. They act by inhibiting one or both of the enzymes responsible for the hydrolysis of acetylcholine in the synaptic cleft [acetylcholinesterase (AChE) and butyrylcholinesterase (BuChE)], thereby increasing available acetylcholine levels and improving neurotransmission. Three cholinesterase inhibitors are commonly used to treat cognitive symptoms in mild-to-moderate AD: rivastigmine (Exelon®; Novartis, Basel, Switzerland), donepezil (Aricept®; Pfizer, New York, NY, USA) and galantamine (Reminyl®/Razadyne®; Johnson & Johnson, New Brunswick, NJ, USA).

Some cholinesterase inhibitors exhibit a dose–response relationship, with higher drug doses correlating with greater enzyme inhibition (1). As AD is a progressive, neurodegenerative disorder where patients deteriorate over time, one goal in clinical practice is to achieve higher doses that maximise the effectiveness of treatment. However, the incidence of adverse events (AEs) associated with oral cholinesterase inhibitors, particularly nausea and vomiting, also increases with higher doses (2). Consequently, achieving and maintaining high therapeutic doses in clinical practice may be difficult.

Rivastigmine is a pseudo-irreversible inhibitor of both AChE and BuChE and has been shown in a number of clinical trials to be efficacious in AD (3–5). Unlike other cholinesterase inhibitors that are only available in oral formulations, a novel transdermal rivastigmine patch has been developed and approved for the treatment of mild-to-moderate AD in many countries worldwide, including the USA, Latin America, Europe and Asia. The pharmacokinetic rationale for the patch suggests that it may provide clinical effectiveness with a more favourable tolerability profile, allowing a simple one-step titration to the recommended dose. The patch design itself may prove more convenient, easier to use and provide visual reassurance that the treatment has been administered, thus potentially improving compliance (6,7).

The rivastigmine patch is the first transdermal treatment for AD, and another cholinesterase inhibitor is also being developed to deliver medication transdermally (8). Patches may have inherent advantages over conventional oral formulations, and it is interesting to consider how they might change the treatment paradigm. This article reviews the clinical features of patch therapy for dementia, compared with conventional oral administration.

## Challenges with orally administered cholinesterase inhibitors

When a drug is administered orally, it is absorbed through the gastrointestinal wall, and plasma drug levels rise rapidly to their peak level (*C*_max_). Drug plasma levels then fall until the next dose is administered, at which point the plasma levels are at their lowest (*C*_min_). Larger and more frequent plasma fluctuations are associated with an increased incidence of cholinergically mediated side effects, particularly gastrointestinal AEs such as nausea and vomiting (2). By reducing *C*_max_ and slowing the time that it takes to reach *C*_max_ (*t*_max_), the potential for these AEs may be reduced (2).

Between *C*_max_ and *C*_min_ lies an optimal therapeutic window, which, because of the dose–response relationship associated with cholinesterase inhibitors, offers the best balance between efficacy and the potential for side effects. The ideal pharmacokinetic profile for a cholinesterase inhibitor treatment would be a smooth, steady delivery of the drug, maintaining plasma levels within this window.

One way this may be achieved is by modifying the dosing regimen, as demonstrated in a recent, randomised, placebo-controlled trial comparing the efficacy and safety of twice daily (bid) vs. three times daily (tid) rivastigmine capsules (9). By administering the same target daily dose of rivastigmine in smaller, more frequent doses, the *C*_max_ and peak–trough difference in plasma concentration were reduced. This was reflected in a lower discontinuation rate because of AEs in the tid group compared with the bid group (10.6% and 16.7%, respectively; placebo = 9.0%). Furthermore, a greater proportion of subjects reached the target dose (12 mg/day) in the tid group vs. the bid group (60% vs. 43% after the 12-week titration phase) and larger treatment effects were observed for the primary efficacy variables. After 26 weeks, a 0.2-point improvement was observed in the tid group on the cognitive subscale of the Alzheimer’s Disease Assessment Scale, and a 0.1-point improvement on the Clinician's Interview-Based Impression of Change plus Caregiver Input (vs. a 1.2- and 0.1-point deterioration in the bid group respectively) (9). This suggests that a smoother pharmacokinetic profile is associated not only with improved tolerability, but may also allow more patients to reach higher doses, potentially improving treatment effectiveness.

An alternative strategy for reducing *C*_max_ and prolonging *t*_max_ (while maintaining the overall drug exposure) is co-administration of rivastigmine with food. This can delay the absorption of the drug from the gut. However, the effect is heavily dependent on the type of food involved, and as such can be unpredictable. Furthermore, simply reducing *C*_max_ alone does not necessarily improve tolerability. A study evaluating the safety and tolerability of an extended-release (ER) formulation of galantamine showed no advantage of ER capsules vs. conventional capsules (10). The *C*_max_ was reduced with the ER formulation. However, the initial rate of absorption was similar to that seen with conventional capsules, resulting in a similar peak–trough difference of ∼ 40 ng/ml over 24 h. This was reflected in a similar incidence of cholinergic AEs (10). The development of a transdermal patch was therefore favoured over an ER formulation for rivastigmine. By providing smooth and continuous delivery of rivastigmine through the skin and directly into the bloodstream, a transdermal patch would lower *C*_max_ and prolong *t*_max_ while maintaining drug exposure. As discussed, this could potentially improve tolerability, allow patients easier access to therapeutic doses and optimise the effectiveness of treatment (6). The dosing frequency study, combined with improved patch technology, therefore provided the rationale for the development of a rivastigmine transdermal patch for the treatment of AD.

## Pharmacokinetic profile of the rivastigmine patch

### Multilayer matrix patch design

Unlike early transdermal patches, which utilised a ‘reservoir’ of the drug dissolved in an adjunct to facilitate drug absorption through the skin (usually alcohol), the rivastigmine transdermal patch uses a modern matrix design. This combines the drug, antioxidants, a polymer mixture that controls the drug delivery rate, and a silicone matrix adhesive to make a single ‘polymeric matrix’ layer. This allows smooth, controlled delivery of the drug via diffusion from the matrix and enables the patches to be kept small, thin and discreet (11).

### Absorption

The efficiency and rate of absorption of a drug through the skin is dependent on many biological and physiochemical factors. Lipophilic agents may pass rapidly through the *stratum corneum* via the lipid-rich intercellular space, whereas hydrophilic agents must dissolve and diffuse through cellular-bound water into the bloodstream (11). The molecular size of the agent is also an important factor in determining the suitability for transdermal delivery; smaller molecules tend to be absorbed faster than larger molecules, with very large molecules such as insulin (5808 Da) being too large to pass through the skin. It has been suggested that any compound with a molecular weight above 500 Da is likely to be unsuitable for transdermal delivery (12). As a small (∼ 250 Da), lipophilic and hydrophilic molecule, rivastigmine is chemically well-suited to transdermal delivery.

To provide the necessary concentration gradient to drive the diffusion process through the skin, all rivastigmine transdermal patches are loaded with a greater amount of rivastigmine than will be absorbed into the bloodstream. In a study of 51 AD patients, the average amount of rivastigmine absorbed from a patch over a 24-h application period was approximately 50% of the total loading dose. The 5 cm^2^ patch released 4.6 mg (51% of 9 mg), the 10 cm^2^ patch released 9.5 mg (53% of 18 mg), the 15 cm^2^ patch released 13.3 mg (49% of 27 mg) and the 20 cm^2^ patch released 17.4 mg (48% of 36 mg) (13). Absorption of any remaining rivastigmine following the 24-h application period was shown to occur very slowly (13). Patients should therefore not be at risk of toxic exposure should a new patch be mistakenly applied without prior removal of the previous patch. Once removed, the short elimination half-life (*t*_1/2_) of rivastigmine (capsule doses = 1.3–1.9 h; 17.4 mg/24 h patch = 3.4 h) (13) ensures the rapid reduction of drug levels in the plasma. As a result, even with the continuous delivery provided with the rivastigmine patch, there is little potential for accumulation in the body.

### Daily pharmacokinetic profile

**Figure 1:**
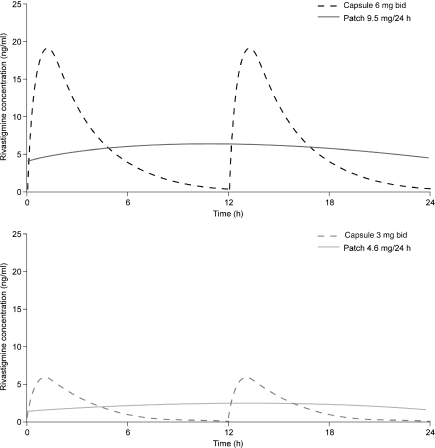
Steady-state rivastigmine plasma levels for a typical patient following administration of the 9.5 mg/24 h rivastigmine patch vs. 6 mg bid capsules, and the 4.6 mg/24 h rivastigmine patch vs. 3 mg bid capsules. The model adjusts for body weight and gender (15)

The results from an open-label study of 51 AD patients randomised to rivastigmine patch (4.6–17.4 mg/24 h; 5–20 cm^2^), or capsules (3–12 mg/day), were used in a compartmental analysis to model rivastigmine plasma levels over a 24-h application period (Figure 1). Drug exposure was assessed by measuring the area under the curve over a 24-h treatment period (AUC_24 h_), using a specific power model (14).

The 4.6 mg/24 h patch was shown to provide comparable rivastigmine exposure to a 6 mg/day capsule dose [AUC_24 h_ = 64 and 60 ng·h/ml (p = ns) respectively] and the 9.5 mg/24 h patch comparable exposure to the highest recommended capsule dose [12 mg/day; AUC_24 h_ = 166 and 207 ng·h/ml (p = ns) respectively]. The 13.3 mg/24 h and 17.4 mg/24 h patches provide greater rivastigmine exposure than any approved oral dose (AUC_24 h_ = 312 and 474 ng·h/ml).

All patch doses provided smoother and more continuous delivery of rivastigmine than oral administration (Figure 1) (15). Both the 4.6 mg/24 h and 9.5 mg/24 h patches provided significantly lower rivastigmine *C*_max_ and longer *t*_max_ (all p < 0.001) vs. capsule doses of comparable exposure: 6 mg/day (*C*_max_ 3.3 vs. 6.8 ng/ml; *t*_max_ 8.2 vs. 1.2 h) and 12 mg/day (*C*_max_ 8.7 vs. 21.6 ng/ml; *t*_max_ 8.1 vs. 1.4 h) respectively (15). These results are supported by a separate, non-compartmental, non-adjusted analysis of the same data (13). Similarly, a recent study comparing rivastigmine oral solution (3 mg/day) with the 9.5 mg/24 h patch showed the patch to have a 20% lower *C*_max_ and 14-times longer *t*_max_, with five-times the drug exposure of the oral solution (*C*_max_ = 5.8 vs. 7.6 ng/ml; *t*_max_ = 14.1 vs. 1.0 h; AUC_∞_ = 118 vs. 23 ng·h/ml respectively) (16).

By providing similar drug exposure with a lower maximum concentration and slower absorption rate, the rivastigmine patch may provide similar efficacy to orally administered rivastigmine, with a more favourable tolerability profile.

### Enzyme inhibition

Rivastigmine is distinct from other available cholinesterase inhibitors (donepezil and galantamine), in that it is a pseudo-irreversible inhibitor of both AChE and BuChE, rather than a rapidly reversible inhibitor of AChE alone. The inhibition of these enzymes *in vivo* was expected to mirror the plasma concentration profiles, with the rivastigmine patch providing steadier and more consistent inhibition than that of oral rivastigmine. AChE has an extremely low concentration in the plasma, and as such the enzymatic activity cannot be measured. However, as rivastigmine is a dual inhibitor, the enzymatic activity of BuChE in the plasma may be used as a marker of target enzyme inhibition over time.

In a pharmacokinetic study by Lefèvre et al. (13), a steady reduction in plasma BuChE activity was observed following patch administration. The target dose 9.5 mg/24 h patch provided maximum inhibition after ∼ 12 h and maintained this for the remainder of the 24-h application period. In contrast, two distinct peak–troughs in plasma BuChE activity were seen with capsule administration. These results indicate that the inhibition of the target enzymes of rivastigmine follows the pharmacokinetic profile closely, with the patch providing smooth and continuous inhibition of plasma BuChE activity over the 24-h application period.

### Application sites

The pharmacokinetic parameters of transdermal drug delivery can vary between patch application sites. The optimal position would offer maximum drug exposure, be easily accessible and avoid adhesion or tolerability issues (e.g. areas of hairy or sensitive skin).

In a recent single-centre, single-dose, open-label, randomised-sequence, application study in 40 healthy men or women aged 40–80 years, the pharmacokinetics, adhesion and skin tolerability of the rivastigmine patch were assessed (17). A 9.5 mg/24 h (10 cm^2^) patch was applied to one of the following five sites and worn for 24 h: upper back, chest, thigh, abdomen and upper arm. Each participant underwent five 24-h applications, one for each application site, which were separated by a 72-h washout period.

**Figure 2:**
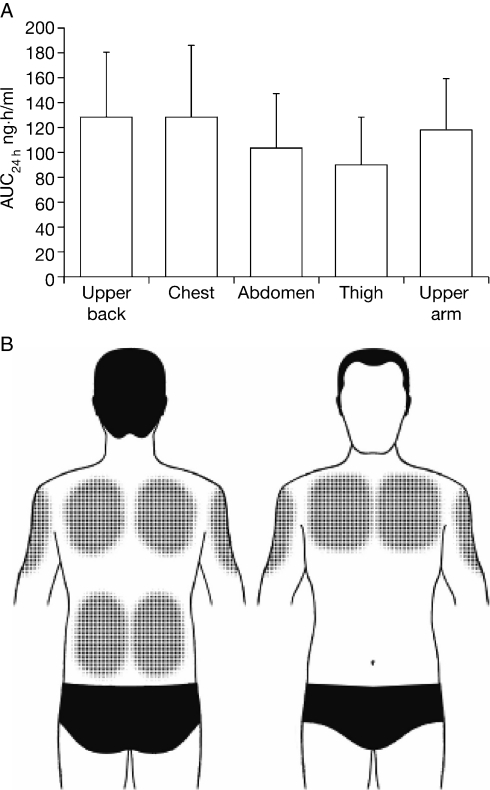
(A) Mean drug exposure (area under curve) following a single 24-h application of the 9.5 mg/24 h rivastigmine patch to the upper back, chest, abdomen, thigh or upper arm of 40 healthy subjects. (B) Recommended application sites

Exposure levels (AUC_24 h_) and *C*_max_ were shown to be the greatest when the patch was applied to the chest, upper back and upper arm (Figure 2; 123, 122 and 116 ng·h/ml respectively). Because of the small molecular size and lipophilic nature of rivastigmine, the minimal skin thickness and subcutaneous body fat at these sites may have contributed to this finding. The degree or level of adhesion was only shown to have a significant effect on AUC_24 h_ and *C*_max_ when the patch was applied to the chest (p = 0.014 and 0.022 respectively). At all application sites, *t*_max_ was very slow (16–22 h) indicating smooth and controlled release of rivastigmine into the bloodstream. Erythema was the only type of skin reaction reported during the study and was least likely to occur when the patch was applied to the upper arm, chest and upper back.

It is therefore recommended that the patch be applied to clean and dry skin on the back, upper arm or chest to obtain maximum rivastigmine exposure with minimal risk of skin reactions. To further reduce the potential for skin irritation, the patch should be alternated daily between sites on the right and the left side of the body.

### Metabolism and bioavailability

Rivastigmine is rapidly and extensively metabolised to the inactive NAP226-90 metabolite by its target enzymes (AChE and BuChE), with little or no interaction with hepatic cytochrome P450 isoenzymes (18). However, a small amount of first-pass metabolism of orally administered rivastigmine may still occur via peripheral cholinesterases in the gut. This effect has been demonstrated in previous pharmacokinetic studies by assessing the ratio of NAP226-90 to rivastigmine following transdermal patch or oral (capsule or solution) administration (13,16). The ratio was shown to be considerably lower following transdermal treatment (0.60–0.72) (13,16) than both capsule (1.10–3.15) (13) and oral solution (3.49) (16) treatment. This indicates that rivastigmine administered transdermally bypasses the phase I metabolism, thereby increasing the systemic bioavailability of the drug. Consequently, the 9.5 mg/24 h rivastigmine patch (10 cm^2^, AUC_24 h_ = 166 ng·h/ml) may provide comparable effectiveness to the highest doses of capsules (12 mg/day, AUC_24 h_ = 207 ng·h/ml), despite having a numerically lower AUC_24 h_.

### Clinical effects with transdermal dosing

One of the major obstacles to the effective treatment of AD with oral cholinesterase inhibitors has been tolerability, which can prevent many patients from reaching efficacious therapeutic doses in clinical practice. Until recently, all cholinesterase inhibitors were administered orally, but the newly developed rivastigmine patch appears to overcome this tolerability obstacle by employing a different dosing route and may offer a substantial clinical advantage.

Modelling analyses adjusting for baseline demographic factors demonstrated that the 9.5 mg/24 h patch (10 cm^2^) provides comparable exposure, and therefore potentially similar efficacy, to the highest doses of rivastigmine capsules (12 mg/day). The pharmacokinetic profile, with a reduced *C*_max_ and prolonged *t*_max_, also predicts an improved tolerability profile vs. conventional rivastigmine capsule administration. These hypotheses are supported by results from the landmark Investigation of transDermal Exelon in ALzheimer’s disease trial (IDEAL). This was a randomised, double-blind, double-dummy, placebo-controlled trial to investigate the efficacy and tolerability of the rivastigmine patch (4.6–17.4 mg/24 h) vs. capsules (3–12 mg/day) in 1195 AD patients (19). Patients randomised to patch treatment were started on the 4.6 mg/24 h patch and titrated in a single step to the recommended 9.5 mg/24 h patch. During the 24-h application period, patients were able to pursue all normal activities, including washing and bathing. The trial was also conducted in countries with varying climates, including some hot and humid regions (e.g. Guatemala, Venezuela).

The 9.5 mg/24 h patch provided similar efficacy to the highest doses of capsules (12 mg/day) on various outcome measures (Table 1), with three times fewer reports of nausea and vomiting (7.2% vs. 23.1% and 6.2% vs. 17.0% respectively; Table 1) (19). This supports the rationale for the patch that a smoother pharmacokinetic profile would yield fewer cholinergically mediated AEs while maintaining therapeutic concentrations. Similar efficacy between the 9.5 mg/24 h patch and 12 mg/day capsule groups, despite the patch providing slightly less drug, demonstrates the advantage with transdermal delivery of the avoidance of first pass metabolism by peripheral cholinesterases in the gut.

**Table 1:** Results from a randomised, double-blind, placebo-controlled trial of 1195 patients with mild-to-moderate AD: mean changes from baseline at week 24 for primary and secondary outcome measures, by double-blind treatment group (9.5 mg/24 h rivastigmine patch, capsule and placebo groups), together with incidences of nausea and vomiting (19)

	Mean 24-week change from baseline		
	9.5 mg/24 h patch	Capsule (3–12 mg/day)	Placebo
**Primary outcomes**			
ADAS-cog	−0.6**	−0.6**	1.0
ADCS-CGIC	3.9**	3.9**	4.2
**Secondary outcomes**			
MMSE	1.1**	0.8**	0.0
ADCS-ADL	−0.1**	−0.5*	−2.3
Trail making test part A	−12.3***	−9.8***	7.7
**Adverse events, %**			
Nausea	7.2	23.1***	5.0
Vomiting	6.2	17.0***	3.3

ITT-LOCF population. MMSE, Mini-Mental State Examination; ADAS-cog, cognitive subscale of the Alzheimer’s Disease Assessment Scale; ADCS-ADL, Alzheimer’s Disease Cooperative Study Activities of Daily Living scale; ADCS-CGIC, Alzheimer’s Disease Cooperative Study Clinical Global Impression of Change. Negative change scores on ADAS-cog and Trail Making Test part A indicate improvement. Negative change scores on MMSE and ADCS-ADL indicate deterioration. ADCS-CGIC is scored as a judgement of change, with 4.0 indicating no change, < 4.0 indicating improvement and > 4.0 indicating deterioration. *p ≤ 0.05, **p ≤ 0.01, ***p ≤ 0.001 vs. placebo; p-values for ADAS-cog, ADCS-ADL and Trail Making Test part A are derived from two-way ANCOVA (explanatory variables: treatment, country and baseline scores), whereas p-values for ADCS-CGIC and MMSE are derived from the CMH van Elteren test using modified ridit scores with country as the stratification variable.

The efficacy of the 4.6 mg/24 h patch was not assessed in the IDEAL trial, however pharmacokinetic data have demonstrated a similar level of exposure to 6 mg/day capsules (64.1 vs. 60.0 ng h/ml respectively), which is considered an effective therapeutic dose (3–5). Also, fewer reports of nausea and vomiting were reported with the starting dose 4.6 mg/24 h patch (1.9% and 0.5%, weeks 1–4), than the starting 3 mg/day capsule dose (3.1% and 2.0%; Novartis, data on file). Therefore, in contrast to the conventional capsule regimen (16-week, four-step titration from 3 to 12 mg/day), patients treated with the rivastigmine patch are initiated on an effective dose with improved gastrointestinal tolerability, and can then be titrated in a single step to the recommended therapeutic dose (9.5 mg/24 h patch) after only 4 weeks.

The improved tolerability profile of the patch also suggests that it may allow patients an easier path to higher doses, thereby enabling patients to stay on and benefit from effective treatment for longer. This is reflected in the greater proportion of patients who achieved their target therapeutic dose in the 9.5 mg/24 h patch group at the end of the study, compared with the 12 mg/day capsule group (95.9% vs. 64.4% respectively). Further investigations of the efficacy and safety of higher doses of rivastigmine (13.3 mg/24 h, AUC_24 h_ = 312 ng·h/ml) are ongoing.

Transdermal administration typically carries with it the risk of additional AEs not associated with oral administration, such as application site skin irritation and sleep disturbances (because of 24-h drug delivery). However, during the IDEAL trial no new safety issues were reported. In addition, the adhesion of the patch was very good, despite patients being permitted to pursue all normal daily activities including bathing and swimming. Skin irritation was actively assessed by the investigator or caregiver, yet most patients experienced ‘no, slight, or mild’ skin irritation (90–98% across all patch doses), with < 2.5% of patients in any treatment group discontinuing because of adverse skin reactions (19). Clinical experience suggests that the most common form of skin irritation is erythema caused by removal of the patch, which normally resolves after a short period of time (20). In the IDEAL trial, the signs or symptoms that were most frequently reported as moderate or severe were erythema (redness; 8% for the 9.5 mg/24 h rivastigmine patch, up to 4% for placebo) and pruritus (itching; 7% for the 9.5 mg/24 h rivastigmine patch, up to 3% for placebo) (19). As stated previously (and in addition to the lower back), Lefèvre et al. (17) demonstrated that the application of the rivastigmine transdermal patch to the upper arm, chest or upper back is least likely to result in the development of erythema. Daily rotation of the application site is recommended in the product label to minimise skin irritation, avoiding the exact same spot for at least 14 days (although consecutive patches may be applied to the same anatomical site).

## Conclusion

Overall, current clinical data support the pharmacokinetic rationale of the rivastigmine patch, demonstrating that the smooth continuous drug delivery provided by transdermal administration translates into comparable efficacy with an improved tolerability profile vs. oral administration. This drug administration route could therefore allow optimal therapeutic dosing, potentially further improving the effectiveness of treatment. The rivastigmine patch may be the optimal way to deliver rivastigmine to treat AD, and may be the first of several transdermal options in the ‘next generation’ of cholinesterase inhibitor formulations.
